# Evaluation of the Effects of Ag, Cu, ZnO and TiO_2_ Nanoparticles on the Expression Level of Oxidative Stress-Related Genes and the Activity of Antioxidant Enzymes in *Escherichia coli*, *Bacillus cereus* and *Staphylococcus epidermidis*

**DOI:** 10.3390/ijms23094966

**Published:** 2022-04-29

**Authors:** Oliwia Metryka, Daniel Wasilkowski, Agnieszka Mrozik

**Affiliations:** 1 Doctoral School, University of Silesia, Bankowa 14, 40-032 Katowice, Poland; 2 Institute of Biology, Biotechnology and Environmental Protection, Faculty of Natural Sciences, University of Silesia, Jagiellońska 28, 40-032 Katowice, Poland; agnieszka.mrozik@us.edu.pl

**Keywords:** *Escherichia coli*, *Bacillus cereus*, *Staphylococcus epidermidis*, metal nanoparticles, oxidative stress-related genes, catalase, peroxidase, superoxide dismutase

## Abstract

Although the molecular response of bacteria exposed to metal nanoparticles (NPs) is intensively studied, many phenomena related to their survival, metal uptake, gene expression and protein production are not fully understood. Therefore, this work aimed to study Ag-NPs, Cu-NPs, ZnO-NPs and TiO_2_-NPs-induced alterations in the expression level of selected oxidative stress-related genes in connection with the activity of antioxidant enzymes: catalase (CAT), peroxidase (PER) and superoxide dismutase (SOD) in *Escherichia coli*, *Bacillus cereus* and *Staphylococcus epidermidis*. The methodology used included: the extraction of total RNA and cDNA synthesis, the preparation of primers for selected housekeeping and oxidative stress genes, RT-qPCR reaction and the measurements of CAT, PER and SOD activities. It was established that the treatment of *E. coli* and *S. epidermidis* with NPs resulted mainly in the down-regulation of targeted genes, whilst the up-regulation of genes was confirmed in *B. cereus*. The greatest differences in the relative expression levels of tested genes occurred in *B. cereus* and *S. epidermidis* treated with TiO_2_-NPs, while in *E. coli*, they were observed under ZnO-NPs exposure. The changes found were mostly related to the expression of genes encoding proteins with PER and CAT-like activity. Among NPs, ZnO-NPs and Cu-NPs increased the activity of antioxidants in *E. coli* and *B. cereus*. In turn, TiO_2_-NPs had a major effect on enzymes activity in *S. epidermidis*. Considering all of the collected results for tested bacteria, it can be emphasised that the impact of NPs on the antioxidant system functioning was dependent on their type and concentration.

## 1. Introduction

The golden age of nanotechnology started in the 1980s, and its industrial breakthrough in the 21st century revolutionised the conventional approach to science and the production of various materials [1]. Nanoparticles (NPs) have gained particular recognition in medical applications due to their antimicrobial properties and the stability required for effective drug delivery systems; inter alia, such solutions are used as implant coatings. For example, the deposition of Ag-NPs on the surface of dental implants as a surface coating can improve the quality and biocompatibility of the implant and at the same time limit the use of conventional antibiotics [2,3].

The extensive use of NPs in many sectors of the economy and their release into various ecosystems have raised global concern about their adverse effects on living organisms, including microorganisms. Presently, intensive research is carried out on reference microbial strains as well as environmental microorganisms in order to understand the comprehensive impact of NPs on their physiological processes and the surrounding environment and to determine the critical points of the observed effects [1,4,5]. According to the existing literature data, the major processes underlying the antibacterial effects of NPs include the disruption of the bacterial cell membrane, the generation of reactive oxygen species (ROS), the penetration of the bacterial cell membrane and the induction of intracellular antibacterial effects, including interactions with DNA and proteins [4,5,6]. Among these processes, the most significant attention has been paid to the generation of oxidative stress by NPs, as it has been proposed to be the leading mechanism of the biological activity of NPs. The catalytic oxidation of cellular components and building materials induced by ROS can disrupt fundamental metabolic processes and lead to cell death [4,6,7]. To mitigate the threat posed by NPs, bacteria have developed a variety of mechanisms to combat oxidative stress and balance ROS levels that lead to cellular toxicity. The literature data show that the presence of scavenging enzymes such as catalase (CAT), peroxidase (PER) and superoxide dismutase (SOD) that control and deplete ROS levels is of key importance in self-defence mechanisms against oxidative stress in bacteria [5,8,9]. However, under stressful conditions, the activity of antioxidants can be disturbed by the presence of NPs, releasing heavy metal ions and ROS and impairing the functioning of enzymes or their synthesis from the initial molecular stages. For example, in a study by Liao et al. [10], proteomic and biochemical analyses have shown that the exposure of *Pseudomonas aeruginosa* to Ag-NPs resulted in the alterations of CAT, PER and SOD activities, which was co-dependent on the generation of ROS and the up-regulation of these proteins. Additionally, such toxicological effects may depend not only on the unique characteristics of the materials tested but also on the standardised concentration. By way of illustration, in the experiment conducted by Choi et al. [11], an increase in CAT and SOD activities in *Escherichia coli* cells treated with Ag-NPs depended on the increase in the Ag-NPs concentration.

Moreover, the literature reports indicate that NPs may exhibit genotoxic properties and cause DNA and RNA damage and disrupt replication and the expression of genetic information in a cell, along with having a mutagenic effect. This mode of action can be attributed to the direct impact of NPs and the released metal ions as well as the indirect effect via the ROS-mediated and induced SOS responses in bacterial cells [6,12,13,14,15]. An interesting property of NPs is their influence on horizontal gene transfer between microorganisms, as they can facilitate this process, inter alia, through the induction of the formation of conjugation pairs or the stimulation of selected genes expression [16,17,18]. Furthermore, it was established that Au-NPs and Ag-NPs could damage DNA through the electrostatic interactions with phosphate groups in the polyanionic backbone of nucleic acids, in addition to the hydrophobic and van der Walls forces interactions of metal ions and to the oxygen and nitrogen atoms present in nucleic acids [13,19,20]. By contrast, RNA is more susceptible to oxidative damage caused by ROS than DNA, and, without RNA-repair mechanisms, important processes such as the regulation of transcriptional activity may be altered [9]. Additionally, despite research on gene expression and protein synthesis in microorganisms exposed to NPs, the greatest emphasis is put on stress-related, virulence and DNA-repair genes, alongside genes involved in the *quorum sensing* system, which play a vital role in the biofilm formation. Such NPs–microorganisms interactions can negatively impact the functioning of microorganisms, e.g., they can lead to disturbances in the biofilm formation and proper functioning of the *quorum sensing* system [19,21,22]. For example, the treatment of *Azotobacter vinelandii* with Ag-NPs resulted in the down-regulation of the *nif*H gene and decreased nitrogenase activity, leading to the inhibition of nitrogen fixation [23].

It is worth underlining that a limited number of studies consider the action of NPs on the gene expression, protein synthesis and metabolism of bacterial cells regarding the antioxidant defence system. The available information in this field shows the influence of various nanostructures regulating the expression of genes encoding individual antioxidants related to the effective utilisation of ROS and genes encoding proteins involved in oxidation-reduction processes. Yan et al. [24] demonstrated that the presence of Ag-NPs in *P. aeruginosa* culture induced oxidative stress and eventuated in the up-regulation of KatA and SodB ROS-related proteins. Similarly, Zhang et al. [17] reported that the relative expression of genes responsible for the ROS production in *Escherichia coli* and *Pseudomonas putida* exposed to CuO-NPs showed an up-regulation of the *sod*A, *sod*B and *kat*E genes. In turn, de Celis et al. [25] only observed a significant up-regulation of *sod*M in *P. aeruginosa* under Ag-NPs and ZnO-NPs treatment compared to other oxidative stress genes. Correspondingly, an up-regulation of genes encoding SOD and a down-regulation of CAT-related genes were revealed in *Deinococcus radiodurans* cells exposed to ZnO-NPs [26].

**Figure 1:**
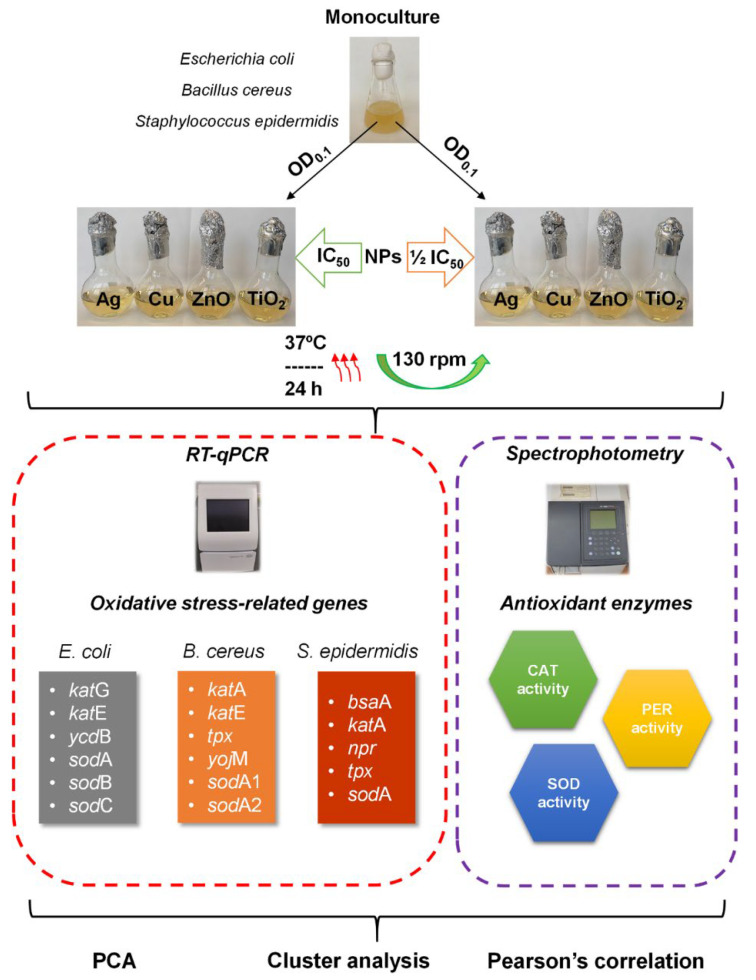
Diagram of experimental set-up.

Due to the scarce information on the direct relationship between the expression level of oxidative stress genes and the activity of the corresponding antioxidant-like proteins in bacteria under NPs stress, it seemed worthwhile to investigate this dependence thoroughly. Therefore, in this experimental study, the intended goals included: (1) assessing and comparing the transcriptional response of model *Escherichia coli*, *Bacillus cereus* and *Staphylococcus epidermidis* to the exposure of Ag-NPs, Cu-NPs, ZnO-NPs and TiO_2_-NPs at a half-maximal inhibitory concentration (IC_50_) and ½IC_50_; (2) measuring the activity of the antioxidant enzymes CAT, PER and SOD and (3) establishing the statistical similarities and differences between measured parameters. An experimental set-up presenting all of the issues to be tested is illustrated in Figure 1.

## 2. Results

### 2.1. Analysis of the Expression Level of Tested Genes in Bacteria under NPs Exposure

Table 1 presents genes encoding antioxidant enzymes in tested bacterial strains chosen for the absolute quantification reaction against selected housekeeping genes used as internal controls. These particular genes were selected by analysing the *E. coli*, *B. cereus* and *S. epidermidis* genomes, as they all encode proteins with CAT, PER and SOD-like activity.

**Figure 2:**
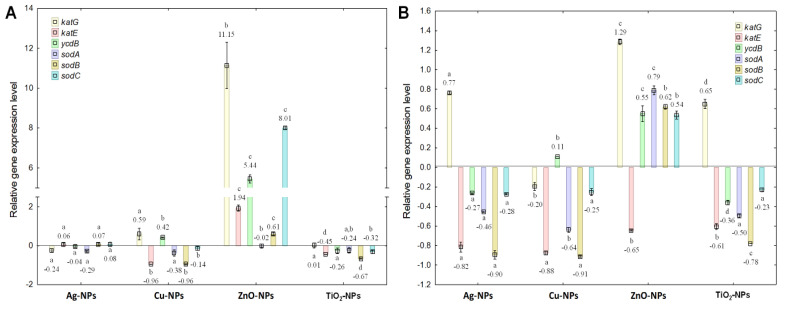
The relative expression levels of *kat*E, *kat*G, *ycd*B, *sod*A, *sod*B and *sod*C genes in *E. coli* exposed to NPs at IC_50_ (**A**) and ½IC_50_ (**B**), measured against *rpo*E as the reference gene (mean ± SD; n = 4). Significant statistical differences (*p* < 0.05) between control and NPs treated cells (n = 4) are represented by different letters by a two-way ANOVA test and followed by a *post-hoc Tukey’s HSD* (honestly significant difference) test.

To thoroughly examine the influence of NPs on the antioxidant profile, the transcriptional response of bacterial cells to NPs was assessed by comparing the relative expression level of selected genes, encoding proteins with CAT, PER and SOD-like activities. Moreover, this study allowed for the determination of correlations between product formation at the expression level of selected genes and the activity of encoded proteins. The obtained results confirmed a significant impact of NPs at IC_50_ and ½IC_50_ on the relative expression level of *kat*E, *kat*G, *ycd*B, *sod*A, *sod*B and *sod*C genes in *E. coli* cells (Figure 2A,B). For example, ZnO-NPs at IC_50_ caused an 11-fold up-regulation of *kat*G and an 8-fold up-regulation of *sod*C. By contrast, when Cu-NPs were added at IC_50_ to *E. coli* culture, there was a 1-fold reduction in the expression level of the *kat*E and *sod*B genes, respectively (Figure 2A). Subsequently, the use of NPs at ½IC_50_ had a divergent effect on the gene expression level compared to their expression induced by IC_50_ (Figure 2B). Overall, the findings showed that Ag-NPs, Cu-NPs and TiO_2_-NPs at ½IC_50_ decreased the expression level of the studied genes, while ZnO-NPs had the opposite effect. The highest 1.3-fold up-regulation was observed for *kat*G in *E. coli* treated with ZnO-NPs. Interestingly, Ag-NPs and Cu-NPs at ½IC_50_ down-regulated the expression of *kat*E and *sod*B (about 1-fold). The statistical analysis uncovered significant differences (*p* < 0.05) for the relative expression levels of *E. coli* genes between NPs treatments at different concentrations. The greatest variation between the results was noted in the data obtained for *kat*E, *kat*G and *sod*A. In turn, ZnO-NPs were found to be the most influential on the transcriptional response of *E. coli* cells. Nevertheless, Ag-NPs, Cu-NPs and TiO_2_-NPs induced considerable but different changes in the expression levels of selected genes. Moreover, changes within the transcriptional response of *E. coli* depended on the concentration and type of NPs used and the kind of gene analysed.

**Figure 3:**
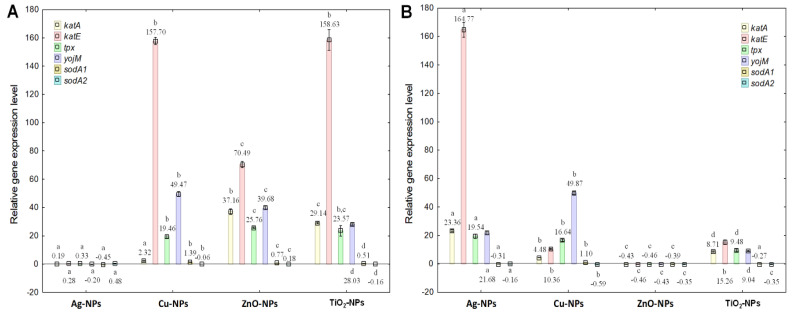
The relative expression levels of *kat*A, *kat*E, *tpx*, *yoj*M, *sod*A1 and *sod*A2 genes in *B. cereus* exposed to NPs at IC_50_ (**A**) and ½IC_50_ (**B**), measured against *rpo*B as the reference gene (mean ± SD; n = 4). Significant statistical differences (*p* < 0.05) between control and NPs treated cells (n = 4) are represented by different letters by a two-way ANOVA test and followed by a *post-hoc Tukey’s HSD* (honestly significant difference) test.

In the case of *B. cereus*, diverse and unique links were noted between the different treatments of cells with individual NPs and the expression levels of *kat*A, *kat*E, *tpx*, *yoj*M, *sod*A1 and *sod*A2 genes (Figure 3A,B). It is worth underlining that the exposure of *B. cereus* to NPs induced a transcriptional response of bacterial cells consisting in up-regulating the expression of the studied genes in most samples. For example, the highest around 159-fold up-regulation of *kat*E was recorded for Cu-NPs and TiO_2_-NPs at IC_50_ (Figure 3A). It is also worth pointing out that ZnO-NPs and Cu-NPs at IC_50_ caused a 40- and 50-fold increase in the expression level of *yoj*M, respectively. Interestingly, the exposure of *B. cereus* to Ag-NPs at ½IC_50_ caused a significant increase (165-fold) in the expression level of *kat*E (Figure 3B). However, a notable decrease (about 0.5-fold) in the expression of all tested genes was confirmed after applying ZnO-NPs to bacterial culture. The results from the statistical analysis proved that all NPs at IC_50_ and ½IC_50_ had a significant and differentiating effect (*p* < 0.05) on the obtained data. The tested NPs mainly exhibited a strong influence on *kat*A and *kat*E genes (*p* = 0.000000). Moreover, it was established that TiO_2_-NPs had the most substantial effect on the expression of all selected genes (*p* = 0.000000). Conclusively, the obtained findings demonstrated varying effects of applied NPs concentrations on the transcriptional response of *B. cereus*. The genes most susceptible to the effects of NPs were related to proteins revealing CAT and PER-like activities.

**Figure 4:**
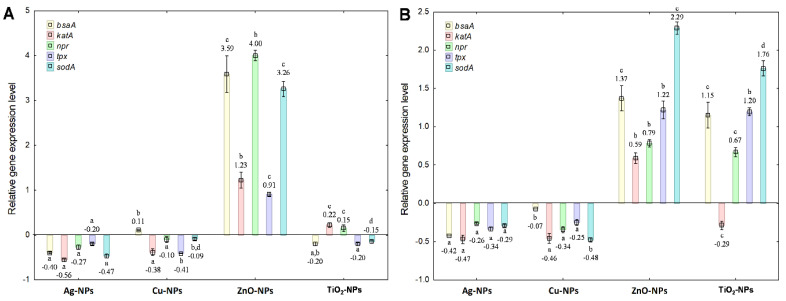
The relative expression levels of *bsa*A, *kat*A, *npr*, *tpx* and *sod*A genes in *S. epidermidis* exposed to NPs at IC_50_ (**A**) and½IC_50_ (**B**), measured against *rpo*B as the reference gene (mean ± SD; n = 4). Significant statistical differences (*p* < 0.05) between control and NPs treated cells (n = 4) are represented by different letters by a two-way ANOVA test and followed by a *post-hoc Tukey’s HSD* (honestly significant difference) test.

In a parallel set of experiments, it was established that the treatment of *S. epidermidis* with NPs at both concentrations significantly altered the transcriptional response of bacterial cells, affecting the expression of *bsa*A, *kat*A, *npr*, *tpx* and *sod*A genes (Figure 4A,B). Predominantly, Ag-NPs, Cu-NPs and TiO_2_-NPs at IC_50_ showed a down-regulation of the tested genes, whereas adding ZnO-NPs caused their up-regulation (Figure 4A). The highest 0.6-fold decrease in the expression level was recorded for the *kat*A gene in the cells exposed to Ag-NPs; however, ZnO-NPs resulted in the most distinctive increase (about 4-fold) in the expression of *bsa*A, *npr* and *sod*A. Compared to the IC_50_ dose, the application of Ag-NPs and Cu-NPs at ½IC_50_ to bacterial culture caused prominent down-regulation of the tested genes, whereas ZnO-NPs and TiO_2_-NPs reflected in their up-regulation. The greatest decrease in the expression of *sod*A (0.5-fold) was confirmed in the cells under Cu-NPs exposure, whereas the greatest increase (2.3-fold) in the expression of this gene occurred in the presence of ZnO-NPs. Statistical analysis revealed significant differences between the treatments of bacterial cells with both NPs concentrations (*p* < 0.05). Furthermore, the highest statistical differences were calculated for the expression of *sod*A after treatment of the bacteria with Ag-NPs (*p* = 0.00027), Cu-NPs (*p* = 0.000001), ZnO-NPs (*p* = 0.000045) and TiO_2_-NPs (*p* = 0.000000). It is worth underlining that TiO_2_-NPs were the most impactful on the tested genes’ expression levels.

Conclusively, significant and different changes in the genes’ expression levels in each bacterial strain under NPs exposure indicate alterations in the product development at the genetic level. It was established that the treatment of *E. coli* and *S. epidermidis* resulted mainly in the down-regulation of tested genes, whilst the up-regulation of targeted genes was observed in *B. cereus* cells. Considering all of the collected data, TiO_2_-NPs caused the greatest differences in the transcriptional response of *B. cereus* and *S. epidermidis*, while in *E. coli*, most significant differences in the relative expression levels of tested genes occurred in the presence of ZnO-NPs. Overall, the conducted analyses confirmed most significant influence of NPs on the expression of genes encoding proteins with PER and CAT-like activity in all strains.

### 2.2. Activity of CAT, PER and SOD in Bacteria Exposed to NPs

**Figure 5:**
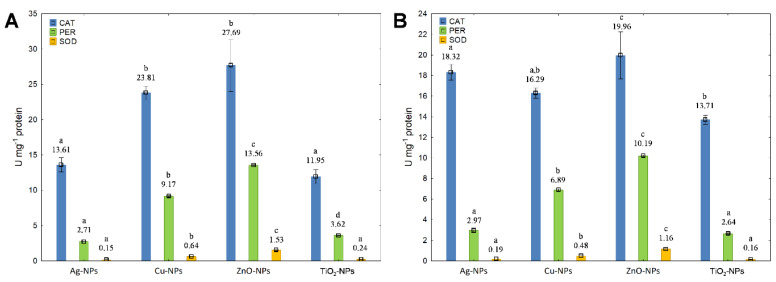
The activity of CAT, PER and SOD in *E. coli* exposed to NPs at IC_50_ (**A**) and ½IC_50_ (**B**) (mean ± SD; n = 3). Significant statistical differences (*p* < 0.05) between control and NPs treated cells (n = 3) are represented by different letters by a two-way ANOVA test and followed by a *post-hoc Tukey’s HSD* (honestly significant difference) test.

In order to examine the functioning of the catalytic antioxidant system in bacteria cells, the activity of the primary antioxidant enzymes, including CAT, PER and SOD, was evaluated. The results obtained for *E. coli* showed a clear dependence of enzymes activity on the concentration of NPs (Figure 5A,B). The treatment of *E. coli* with ZnO-NPs and Cu-NPs at IC_50_ resulted in the highest increase in CAT activity (Figure 5A). A similar increase in CAT activity was observed in bacteria exposed to NPs at ½IC_50_, with ZnO-NPs and Ag-NPs having the most substantial impact on its catalytic activity (Figure 5B). Interestingly, the exposure of *E. coli* to Ag-NPs and TiO_2_-NPs at ½IC_50_ caused a higher (about 44% and 16%) increase in CAT activity compared to its activity at IC_50_. Furthermore, analysis of the variance of changes in CAT activity between individual treatments revealed significant statistical differences for Ag-NPs, Cu-NPs, ZnO-NPs and TiO_2_-NPs at IC_50_ and ½IC_50_ since *p*-values (0.0029, 0.00026, 0.036 and 0.045, respectively) did not exceed the significance level of α = 0.05. By comparison, the stimulating effect of individual NPs at IC_50_ on PER activity in *E. coli* can be illustrated as follows: Ag-NPs < TiO_2_-NPs < Cu-NPs < ZnO-NPs (Figure 5A). The highest increase in PER activity occurred in the presence of ZnO-NPs at IC_50_. Intriguingly, Cu-NPs, ZnO-NPs and TiO_2_-NPs at ½IC_50_ caused a smaller increase in PER activity than these NPs at IC_50_ (Figure 5B). It is worth noting that all NPs except for Ag-NPs resulted in significant differences between dosage treatments (*p* < 0.05). In corresponding experiments relating to the measurements of SOD activity in *E. coli* exposed to NPs, a stimulating trend of individual NPs at both concentrations was established (Figure 5A,B). The highest increase in SOD activity was documented in the presence of ZnO-NPs at IC_50_ (Figure 5A). An equally high increase in its activity was also established for ZnO-NPs at ½IC_50_ (Figure 5B). Regarding the statistical significance between SOD functioning in *E. coli* cells under different experimental conditions, it was confirmed that only TiO_2_-NPs did not result in significant differences (*p* > 0.05). Summarising this series of studies, it can be concluded that, among the studied NPs, the most substantial impact on the activity of antioxidant enzymes in *E. coli* showed ZnO-NPs and Cu-NPs.

**Figure 6:**
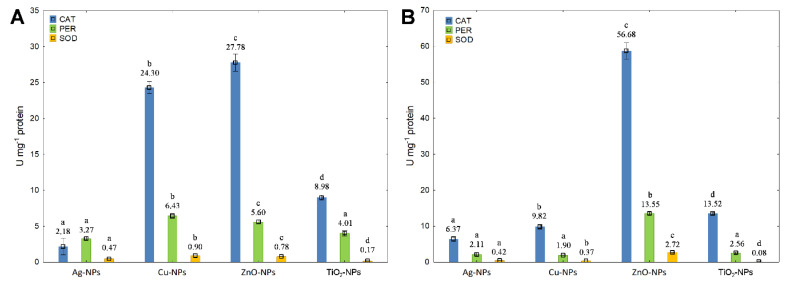
The activity of CAT, PER and SOD in *B. cereus* exposed to NPs at IC_50_ (**A**) and ½IC_50_ (**B**) (mean ± SD; n = 3). Significant statistical differences (*p* < 0.05) between control and NPs treated cells (n = 3) are represented by different letters by a two-way ANOVA test and followed by a *post-hoc Tukey’s HSD* (honestly significant difference) test.

In a parallel experiment, the activity of antioxidant enzymes in *B. cereus* exposed to NPs was measured. The effect of individual NPs at IC_50_ on the increase in CAT activity may be illustrated as follows: Ag-NPs < TiO_2_-NPs < Cu-NPs < ZnO-NPs (Figure 6A). A relatively high stimulation of CAT activity was recoded for Cu-NPs and ZnO-NPs. Interestingly, the activity of CAT also increased after treatment with Ag-NPs (by 192%), TiO_2_-NPs (by 51%) and ZnO-NPs (by 104%) at ½IC_50_, whilst Cu-NPs caused its decrease (by 60%) compared to its activity at IC_50_ (Figure 6B). Similar to CAT, PER of *B. cereus* was most affected by Cu-NPs and ZnO-NPs at IC_50_, reflecting in the increase in its activity (Figure 6A). Furthermore, Cu-NPs at IC_50_ proved to have a greater impact on the activity of PER than Ag-NPs and TiO_2_-NPs. Predictably, Ag-NPs, Cu-NPs and TiO_2_-NPs added to the bacteria cultures at ½IC_50_ had a lower stimulating effect on PER activity than these NPs at IC_50_ (Figure 6A,B). Based on the measurements of SOD activity, it can be concluded that Cu-NPs and ZnO-NPs at IC_50_ had the greatest impact on the stimulation of this enzyme activity (Figure 6A). Interestingly, the treatment of *B. cereus* with ZnO-NPs at ½IC_50_ in contrast to IC_50_ resulted in a higher increase in SOD activity by 249% (Figure 6B). It is worth underlining that the obtained findings for CAT, PER and SOD activities in *B. cereus* proved to be statistically significant for both concentrations of all NPs (*p* < 0.05). Overall, the greatest changes in the activity of assayed enzymes in cells treated with NPs at IC_50_ and ½IC_50_ were observed for CAT and SOD. On the other hand, the greatest differentiation of the overall enzymatic activity in bacterial cells exposed to NPs at IC_50_ and ½IC_50_ was observed in the presence of Cu-NPs and ZnO-NPs.

**Figure 7:**
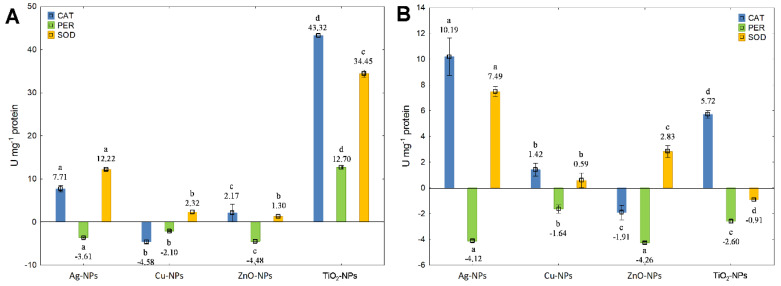
The activity of CAT, PER and SOD in *S. epidermidis* exposed to NPs at IC_50_ (**A**) and ½IC_50_ (**B**) (mean ± SD; n = 3). Significant statistical differences (*p* < 0.05) between control and NPs treated cells (n = 3) are represented by different letters by a two-way ANOVA test and followed by a *post-hoc Tukey’s HSD* (honestly significant difference) test.

In the case of *S. epidermidis*, it is difficult to indicate the similarities in the influence of individual NPs on the activity of CAT, SOD and PER due to their very diverse and often contradictory effect on the activity of tested enzymes. Since each NPs had a different effect on the antioxidant activity profile, it was found that the presence of Cu-NPs (IC_50_) caused a decrease in CAT activity, whilst other treatments resulted in the stimulation of CAT functioning (Figure 7A). The greatest increase in CAT activity was recorded for TiO_2_-NPs at IC_50_. Comparatively, TiO_2_-NPs at ½IC_50_ had a smaller stimulating effect on CAT activity by 87% than at IC_50_ (Figure 7B). In turn, an opposite effect of Cu-NPs and ZnO-NPs at both concentrations on CAT activity was documented. Moreover, significant differences in CAT activity at IC_50_ and ½IC_50_ were established for Cu-NPs (*p* = 0.00011), ZnO-NPs (*p* = 0.028) and TiO_2_-NPs (*p* = 0.000003), except for Ag-NPs (*p* = 0.058). Simultaneously, the exposure of *S. epidermidis* to NPs at both concentrations reduced PER activity, except for TiO_2_-NPs at IC_50_ generating the increase in its activity (Figure 7A,B). The high decrease in PER activity occurred in the cells exposed to ZnO-NPs at IC_50_ and ½IC_50_. Statistical analysis revealed significant differences in the activity of PER treated with Ag-NPs (*p* = 0.0046), ZnO-NPs (*p* = 0.014) and TiO_2_-NPs (*p* = 0.000001), except for Cu-NPs (*p* = 0.088). The conducted research also confirmed an enhancing impact of NPs on SOD activity in *S. epidermidis* (Figure 7A,B). The highest increase in SOD activity was ascertained for Ag-NPs and TiO_2_-NPs at IC_50_. Interestingly, the treatment of bacteria with TiO_2_-NPs at ½IC_50_ caused a significant decrease in SOD activity by 103% (Figure 7B). It is worth pointing out that both Ag-NPs and Cu-NPs exhibited a smaller inhibiting effect on SOD activity at ½IC_50_ than at IC_50_. Moreover, the obtained findings for SOD activities were demonstrated to be statistically significant for both concentrations of all NPs (*p* < 0.05).

Conclusively, considering all of the collected results for *E. coli*, *B. cereus* and *S. epidermidis*, it can be emphasised that the impact of NPs on the antioxidant system functioning was dependent on their type and concentration. Furthermore, *E. coli* and *B. cereus* under NPs exposure were characterised by increased activity of antioxidants, mainly affected by ZnO-NPs and Cu-NPs. In turn, TiO_2_-NPs had a major effect on enzymes activity in *S. epidermidis*. More significant differences in enzymes activity were found for *B. cereus* and *S. epidermidis* than for *E. coli*. The obtained findings indicated different degrees of sensitivity and susceptibility of the tested strains to varying concentrations of NPs.

### 2.3. Statistical Data Exploration

**Figure 8:**
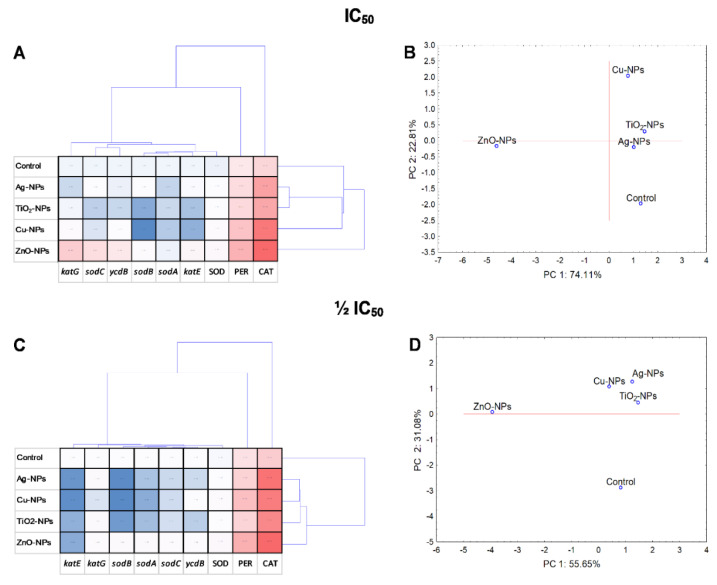
Projection of cluster analysis dendrograms (**A**,**C**) and PCA analysis biplots (**B**,**D**) for *E. coli* exposed to NPs at IC_50_ and ½IC_50_.

**Figure 9:**
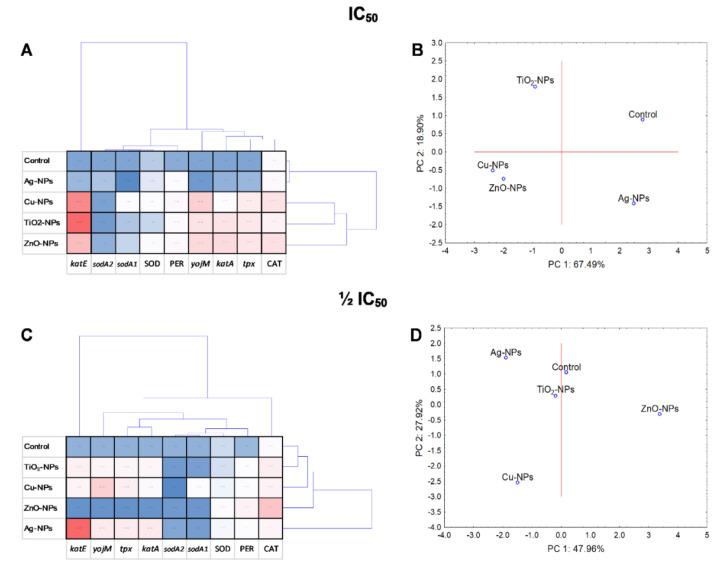
Projection of cluster analysis dendrograms (**A**,**C**) and PCA analysis biplots (**B**,**D**) for *B. cereus* exposed to NPs at IC_50_ and ½IC_50_.

**Figure 10:**
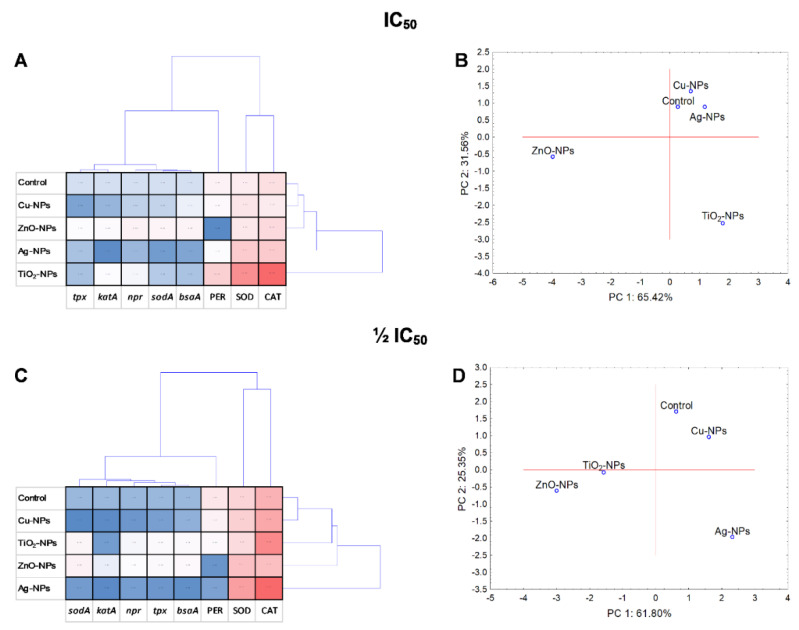
Projection of cluster analysis dendrograms (**A**,**C**) and PCA analysis biplots (**B**,**D**) for *S. epidermidis* exposed to NPs at IC_50_ and ½IC_50_.

Statistical analyses including PCA and cluster analysis were performed to evaluate the NPs treatment variability and variance between the whole set of data. At the same time, a cluster analysis of all variables was carried out in order to check whether the examined variables are statistically related to each other and whether there are correlations in the collected data. Additionally, the interdependence of the tested components and the NPs-concentration was included. PCA analyses and a coordination biplot for *E. coli* distinguished two clusters along PC1, separating ZnO-NPs as the most differentiating NPs (Figure 8B,D). Two clusters along PC1 were also created for *B. cereus*, including Cu-NPs with ZnO-NPs, Ag-NPs with control and separate for TiO_2_-NPs (Figure 9B,D). Similar results to *E. coli* were obtained for *S. epidermidis* (Figure 10B,D).

The performed cluster analysis displayed a correlation of results specific for each strain. For example, the diagram generated for *E. coli* exposed to NPs revealed that the most differentiating were ZnO-NPs, while Ag-NPs and TiO_2_-NPs had a comparable impact on bacteria (Figure 8A,C). Additionally, it was demonstrated that PER and CAT activity and the expression level of *kat*G in *E. coli* had a major discriminating influence on the obtained data. It is worth pointing out that a strong positive correlation (*p* < 0.05) was validated for PER with CAT (*r* = 0.950) and *kat*G with SOD (*r* = 0.941), *kat*E (*r* = 0.945), *ycd*B (*r* = 0.998) and *sod*C (*r* = 0.997) (Table S1). Regarding *B. cereus*, the obtained dendrogram projection revealed two separate groups for IC_50_. The first included the control and Ag-NPs, while the second consisted of other NPs treatments (Figure 9A). It was documented that ZnO-NPs along with *kat*E, PER and *yoj*M had the most differentiating influence on the data set. Additionally, notable positive relationships (*p* < 0.05) were only observed for *yoj*M with CAT (*r* = 0.929), PER (*r* = 0.882), *tpx* (*r* = 0.892) and *sod*A1 (*r* = 0.96) and for PER with SOD (*r* = 0.888) (Table S2). The dendrogram created for *S. epidermidis* showed the formation of two groups, one dedicated to TiO_2_-NPs and the other containing other treatments (Figure 10A). It was proved that all analysed enzymes were the most differentiating variables, without correlation (*p* < 0.05) with the relative genes’ expression levels (Table S3).

The PCA and cluster analyses revealed that the dose of NPs had a significant impact on the oxidative system of *B. cereus* and *S. epidermidis*, especially in the case of TiO_2_-NPs, Ag-NPs and ZnO-NPs. Conversely, the results from PCA for *E. coli* showed that IC_50_ and ½IC_50_ NPs concentration had a slight effect on the enzymes activity and genes expression.

## 3. Discussion

In recent years, advances in nanotoxicological studies show that both the intentional and unintentional exposure of living organisms to NPs force them to overcome toxicological effects and thus precisely model biological activity profiles. The often diverse experimental data on the cytotoxicity of various NPs present a challenge to the scientific community due to the complexity and difficulty of verifying the extremely complex microorganism–NPs interactions. As each type of NPs causes a different cellular bacterial response, various assays and research methods are critical in tracking NPs-induced changes at the molecular level. Understanding these changes is essential to ensuring their safe application and to defining their impact when released into various ecosystems [27,28,29].

In the conducted studies, the combination of transcription and enzymatic analyses allowed for a new and in-depth assessment of the expression level of genes encoding antioxidant enzymes and other related products, together with the precise determination of the correlation and relationship between these intracellular processes in model *E. coli*, *B. cereus* and *S. epidermidis* strains. Such an approach to the studied topic revealed that the influence of NPs on the analysed phenomena depended on their type and current concentration in the bacterial culture. It is worth underlining that the concentration of NPs was not always associated with the higher impact of NPs on the tested processes. For example, Ag-NPs, Cu-NPs and TiO_2_ at ½IC_50_ appear to be more toxic to tested bacteria than at IC_50_. Such a dose-dependency was also documented by Leung et al. [30], who established that TiO_2_-NPs at a lower concentration had greater influence on the expression of ROS-related proteins than at a higher concentration. The divergent effects of NPs at different concentrations on bacterial cells may be related to their bioavailability depending on the ability of these structures to agglomerate/aggregate, changing their direct contact with the cell. In this work, this is clearly shown by the opposite results obtained in the same analyses for selected strains and NPs. For example, an up-regulation of *kat*E and *kat*A genes in *E. coli* and *S. epidermidis* occurred after exposure to ZnO-NPs and TiO_2_-NPs at IC_50_, respectively; however, an opposite effect was observed at ½IC_50_. NPs have different and more effective properties than their larger counterparts. It is worth emphasising that there are no identical NPs at the atomic level [31,32]. Furthermore, it has been observed that the size of NPs affects their various properties, including toxicity, which causes various functional changes in the cell [31]. Here, no size-toxicity of NPs was observed, while the main factors influencing the variability of the obtained results could be the rather different atomic structures, composition and dosage of the tested NPs. This is justified by the toxicity of the heavy metals themselves, because, for example, Cu and Ag have been used since ancient times as antibacterial agents [33,34]. However, they may show different toxicological properties as NPs compared to their ionised form. Confirming this possibility, Peszke at al. [35] reported that Cu as Cu/SiO_2_ nanocomposite (NCs) was more toxic to *E. coli*, *Pseudomonas putida* and *Arthrobacter globiformis* than Cu ions; however, an opposite effect was observed for Ag ions and Ag/SiO_2_-NCs. These results further confirm that the toxicity of NPs depends on their aggregation/agglomeration and the release of bioavailable metal ions.

Previous studies have suggested that the presence of NPs in bacterial cultures may influence their antioxidant activity and the transcription of stress-related genes responsible for protecting bacterial cells from oxidative stress [30,36]. Herein, the presented results confirmed the diversified changes in these cellular processes, depending on the strain tested. In the case of *E. coli*, it was established that the transcriptional activity of *kat*G and *kat*E genes encoding CAT and PER-like proteins was affected by different NPs treatments. The changes in the transcriptional activity of these genes were directly proportional to the overall activity of CAT and PER enzymes, especially in the presence of ZnO-NPs and Cu-NPs at IC_50_. It should be emphasised that the activity of CAT in *E. coli* was higher in all samples than PER activity, even in the control conditions. This phenomenon may be explained by the fact that in *E. coli* are present two types of CAT: hydroperoxidase I (HPI) and hydroperoxidase II (HPII), with bifunctional CAT-PER and monofunctional CAT-like activities, respectively [37,38]. The level of HPI is regulated by the expression of *kat*G, which is induced by H_2_O_2_. By contrast, *kat*E encoding stable HPII is constitutively expressed, independently of H_2_O_2_ [37,38,39]. Considering this, the increase in CAT activity in *E. coli* cells treated with NPs may result from stimulated HPI activity dependent on the presence of H_2_O_2_. This is in agreement with our previous findings, which revealed that the tested NPs, especially ZnO-NPs and Cu-NPs, induced the formation of H_2_O_2_ along with other types of ROS in bacterial cells [40]. Additionally, a positive correlation between CAT and PER activity along with the expression of *kat*G and *kat*E genes was confirmed by statistical analysis. Moreover, a lower stimulation of SOD activity compared to the CAT and PER activity can be attributed to the down-regulation of genes encoding corresponding proteins. On the contrary, the considerable up-regulation of *sod*C in *E. coli* treated with ZnO-NPs at IC_50_ correlated with greater activity of SOD. This may be explained by the relatively high concentration of Zn^2+^ ions in the cells, this being one of the cofactors of the encoded enzyme. This association was further explained by the positive correlation between SOD general activity and the expression of *sod*C. Although many tested genes in *E. coli* cells were down-regulated, it was established that this did not affect the activity of the antioxidant defence system under NPs stress. It is worth pointing out that mRNA abundance present in a cell at a given time is not always complementary to protein quantity [41]. Considering other mechanisms of the biological activity of NPs, it can be hypothesised that the observed down-regulation of genes encoding antioxidants may be attributed to the increased regulation of genes related to the repair of bacterial outer layers or nucleic acids, as well as genes necessary to maintain cell homeostasis [41].

Conversely, the exposure of *B. cereus* cells to NPs resulted in the up-regulation of most examined genes, corresponding with increased overall activity of CAT, PER and SOD. This may suggest the rapid response of bacterial cells to the stressful conditions caused by the presence of NPs. For example, a relatively high up-regulation of *yoj*M was correlated with elevated SOD activity. This is particularly true for ZnO-NPs and Cu-NPs treatments, because the SOD-like protein encoded by *yoj*M uses Zn^2+^ as a cofactor and can bind Cu^2+^, which in high concentrations enhances the enzyme activity. Furthermore, *sod*A1 in *B. cereus* is constitutively expressed, whilst *sod*A2 expression depends on the growth stages of bacteria together with intracellular O_2_^∙^^−^ concentration [42,43,44]. This explanation is consistent with our previous study because it links the up-regulation of *sod*A2 by Ag-NPs and ZnO-NPs at IC_50_ with the formation of O_2_^∙^^−^ [40]. Similarly, the increased relative expression level of *kat*E in *B. cereus* under Cu-NPs exposure was associated with a stimulation of CAT activity. The *kat*A gene encodes vegetative CAT and *kat*E encodes σ^B^-dependent CAT characterised by different transcriptional activity under stress conditions. In a study by Ganesh Babu et al. [45], the expression of *kat*E in *B. cereus* exposed to AgNO_3_ was directly induced by Ag^+^ ions. This may also be the reason why Ag-NPs at ½IC_50_ caused a high up-regulation of *kat*E. Interestingly, the transcriptional activity of this gene was not reflected in accelerated CAT activity. The reason may be the inactivation of the protein or the inhibition of its synthesis at the translation level through the prevention of tRNA binding to a small ribosome subunit [6,46]. The obtained findings for *B. cereus* revealed that an increase in the transcriptional response of bacterial cells was associated with increased antioxidant function.

Contrary to the strains described above, the experimental data collected for *S. epider-midis* showed significant discrepancies between the expression levels of the studied genes and the activity of their molecular counterparts. Despite these differences, statistical analyses showed a positive correlation between all of the results. Generally, the increase in the expression of selected genes was positively correlated with the activity of the antioxidant enzymes, while a down-regulation usually had the opposite effect. For example, a relatively high up-regulation of *kat*A in the cells treated with ZnO-NPs at ½IC_50_ was not reflected in the stimulation of CAT activity. Gene *kat*A encodes CAT and is regulated by Fe^2+^ [47]. Zn^2+^ and Cu^2+^ cations can form very stable structures with proteins and bind to protein sites that are not their characteristic binding sites, such as Fe-S clusters [48]. Therefore, it can be assumed that the free metal ions may interact with protein groups (-SH, -NH_2_, -COOH) and Fe-S centres, causing their inactivation [6,49]. Additionally, disturbances in metal homeostasis and H_2_O_2_ detoxification can lead to the inactivation of Fe-dependent enzymes through the oxidation of Fe^2+^ to Fe^3+^ and its dissociation, leaving an open site for Zn^2+^ attachment [50]. It is worth underlining that, generally, the down-regulation of *bsa*A, *npr* and *tpx* in *S. epidermidis* was correlated with relatively low overall activity of PER-like proteins; however, the opposite dependency was documented for ZnO-NPs. A PER-like protein encoded by *tpx* contains a disulphide bond in the structure, which may be a target for the negative effect of ZnO-NPs. Previous studies have shown that metal oxide NPs such as Fe_3_O_4_@Au-NPs had a strong affinity to the protein’s disulphide bonds, altering the functioning of the bacterial redox system [51]. In turn, the expression of *sod*A in *S. epidermidis* is regulated by intracellular and extracellular levels of O_2_^∙^^−^ [52]. In this study, the high SOD activity and the high expression of the corresponding gene in the cells cultured with TiO_2_-NPs (IC_50_) were closely related to the high intracellular concentration of O_2_^∙^^−^ documented in our previous study [40].

The number of works linking the antioxidant activity of bacteria with the expression of the relevant genes is almost invisible. This is because most research focuses on analysing gene expression and the accompanying desired cytotoxic changes in microbial cells. The research findings published so far concerned the changes in the expression level of selected genes belonging to designated categories, including biological processes (e.g., fatty acid metabolic processes), stress responses (e.g., oxidative stress, osmotic stress) and genetic information processing (e.g., DNA repair) [30,36,41,53]. Since no direct correlation between the expression of genes related to oxidative stress and changes in the antioxidant defence system has been experimentally confirmed, comparing studies at the molecular level is a big challenge. Notwithstanding this, Sohm et al. [41] performed a global transcriptomic and proteomic analysis combined with chemical and biochemical analyses for *E. coli* exposed to TiO_2_-NPs. Among 1702 analysed genes, 152 were found to be differentially expressed, with 68 up-regulated and 84 down-regulated. Interestingly, the transcript level of *sod*C encoding SOD (Cu-Zn) was increased by 1.5-fold. In another study by Moore et al. [36], the treatment of *E. coli* with CuO-NPs resulted in a significant 3.4-fold increase in *sod*A expression and other oxidative stress genes. Similarly, the exposure of *Campylobacter jejuni* to ZnO-NPs had a stimulating effect on *kat*A and *sod*B expression, causing their 6-fold and 2-fold increase, respectively [54]. By comparison, in this study, a down-regulation of *sod*A and *sod*C was revealed in *E. coli* treated with Cu-NPs and TiO_2_-NPs at IC_50_ and ½IC_50_. In studies on the effects of ZnO-NPs and TiO_2_-NPs on *E. coli*, Leung et al. [30] found that although ZnO-NPs up-regulated genes associated with ROS-related proteins, they had lower antimicrobial activity compared to TiO_2_-NPs, indicating their opposite effect on gene transcription activity. Additionally, the authors observed a dose-dependent effect, similar to that presented in this paper. For example, ZnO-NPs and TiO_2_-NPs at a higher concentration decreased the expression level of the thiol peroxidase gene, while a lower dose increased its expression.

## 4. Materials and Methods

### 4.1. Bacterial Strains, Nanoparticles and Culture Conditions

This study was conducted using three model bacteria strains, *Escherichia coli* (ATCC^®^ 25922™), *Bacillus cereus* (ATCC^®^ 11778™) and *Staphylococcus epidermidis* (ATCC^®^ 12228™), equipped from American Type Culture Collection (ATCC). The microorganisms were cultured in lysogeny broth (LB mix; tryptone 10 g L^−1^, NaCl 10 g L^−1^, yeast extract 5 g L^−1^) under exposure to four types of nanoparticles, Ag-NPs (cat. 576832, Sigma-Aldrich, <100 nm), Cu-NPs (cat. 774081, Sigma-Aldrich, 25 nm), TiO_2_-NPs (cat. US1019F, US Research, 20 nm) and ZnO-NPs (cat. 677450, Sigma-Aldrich, <50 nm), at a half-maximal inhibitory concentration (IC_50_) and at a concentration equal to half IC_50_ (½IC_50_) (Table 2). The controls were bacterial cells not treated with NPs. Prior to genetic analysis, the bacteria were grown for 4–5 h at 37 °C and under shaking conditions (140 rpm) until they reached the logarithmic growth phase; however, in biochemical tests aimed at measuring the activity of the antioxidant enzymes catalase (CAT), peroxidase (PER) and superoxide dismutase (SOD), the bacteria were cultivated for 24 h to achieve substantial enzyme production.

### 4.2. Extraction of Total RNA and cDNA Synthesis

To isolate the total RNA, the bacterial cultures treated with NPs were centrifuged at 5000 rpm and 4 °C for 25 min. The supernatant was suspended, and the remaining precipitate was washed three times with sterile Millipore water, each time centrifuging the probe content at 14,000 rpm and 4 °C for 10 min. The precipitate after the final wash was used for the extraction of total RNA from the bacterial cells using a GeneMATRIX Universal RNA Purification Kit (cat. E3598, EURx, Gdańsk, Poland). For Gram-positive bacteria, an additional incubation with lysosome at 37 °C for 1 h was performed in order to disintegrate the double-layer of peptidoglycan of the cell wall. The extracted total RNA was subjected to additional purification with RNase-free DNase (Invitrogen, ThermoFisher Scientific, Waltham, MA, USA) to digest the residual genomic DNA present in the samples [55]. The concentration and purity of collected RNA were assessed using an ND-1000 NanoDrop spectrophotometer (ThermoFisher Scientific, Waltham, MA, USA) through the measurement of the absorbance of acquired samples at 230, 260 and 280 nm and the calculating of 260/280 and 260/230 optical density (OD) ratios [36,41,55]. Moreover, the quality and integrity of the obtained RNA samples were examined through agarose gel electrophoresis [55].

The synthesis of cDNA templates was carried out in triplicates using the RevertAid First Strand cDNA Synthesis Kit (cat. K1621, ThermoFisher Scientific, Waltham, MA, USA). For this purpose, 1 µg of total RNA from each sample was used [55]. Aliquots of cDNA were stored at −21 °C for further experiments.

### 4.3. Preparation of Primers

The specific primers for the tested genes were designed using the Primer-BLAST designing tool (https://www.ncbi.nlm.nih.gov/tools/primer-blast/ accessed on 25 March 2022), and genome nucleotide sequences are available for each strain at the ATCC site (https://genomes.atcc.org/genomes/ accessed on 25 March 2022) (Table 1). Each primer pair was designed to have the optimal sequence of 20 nucleotides with ≥50% of GC pairs and ≥60 °C melting temperature (T_m_) to provide the high specificity of starter annealing to the cDNA template in the mainstream reaction [56]. The specificity of the designed primers was initially tested using control reaction with Color Taq PCR Master Mix (2x) (cat. E2525, EURx, Gdańsk, Poland) to eliminate incorrectly matched primers to the generated cDNA template.

### 4.4. Study of the Expression Level of Genes Encoding Antioxidant Proteins

The expression of oxidative stress genes was assessed through the RT-qPCR reaction using a LightCycler^®^ 480 SYBR Green I Master (cat. 04707516001, Roche, Basel, Switzerland). The analysis was carried out in 96-well Multiwell plates in two biological and three technical replicates [55]. The fluorescence signal from the tested samples was measured using a LightCycler^®^ 96 Real-Time PCR System (Roche, Basel, Switzerland) under the following experiment set-up: preincubation at 95 °C for 10 min, 3 step amplification in 45 cycles consisting of 95 °C—10 s, 60 °C—10 s and 72 °C—10 s, melting at 97 °C—1 s, 65 °C—60 s and 95 °C—10 s and cooling at 40 °C for 10 s [55]. The results from the melting curve assays were used as supplementary data for checking the specificity of the amplification reactions [36,41]. Furthermore, RT-qPCR’s efficiency was examined by preparing standard curve quantification of the serial dilution of the cDNA control template and each primer pair. The efficiency of the RT-qPCR reaction was calculated by a qPCR Efficiency Calculator provided by ThermoFisher Scientific (https://www.thermofisher.com/pl/en/home/brands/thermo-scientific/molecular-biology/molecular-biology-learning-center/molecular-biology-resource-library/thermo-scientific-web-tools/qpcr-efficiency-calculator.html/ accessed on 25 March 2022). To determine the level of relative expression of the studied genes, the method employed by Livak and Schmittgen [57] was used. The reference genes used as an internal control for *E. coli* included: *gyr*A, *gyr*B and *rpo*E [53,58]. A similar set of primary metabolic genes was chosen for *B. cereus* strains except for *rpo*E, replaced by *rpo*B [59]. Contrarily, the housekeeping genes of *S. epidermidis* comprised *gyr*B, *pyk* and *rpo*B genes [60]. These genes were used for normalisation against target genes due to a similar level of expression in both treated and untreated bacterial cells [36,41].

### 4.5. Determining the Activity of CAT, PER and SOD

To compare the changes in the product formation at the expression level of selected genes, the activity of their secondary molecular equivalents, CAT, PER and SOD, was assessed. The activity of all enzymes was measured in crude enzyme fraction obtained from bacterial cells exposed to NPs using Hegeman’s method [61]. The CAT activity was measured by observing a decrease in the absorbance at λ = 240 nm in time, equivalent to the H_2_O_2_ degradation by an active enzyme [62,63]. The activity of PER was determined by the enzyme assay provided by Sigma-Aldrich, where an increase of absorbance at λ = 420 nm, specific to an increase in the colourful purpurogallin product in time, was recorded. To assess SOD activity, a commercial kit with xanthine oxidase and tetrazolium salt as reagents (cat. 19160, Sigma-Aldrich, St. Louis, MI, USA) was used. The absorbances measured at λ = 450 nm were used in the SOD activity calculations according to Zhang et al. [64]. The protein concentrations in the isolated protein fractions were determined by the Bradford method [65], and, finally, CAT, PER and SOD activities were presented as U · mg^−1^ of protein.

### 4.6. Statistical Analysis

All of the experimental data were presented as the mean ± the standard deviation (SD) of four replicates. Grubbs’ outlier test was applied to all experimental data to verify and exclude any significant outliers from the results. The statistical significance between studied NPs, their effect on enzymes activities and the relative expression levels of the selected genes was followed up using a one-way ANOVA. The experimental groups were separated by applying the post-hoc Tukey’s honest significant difference test (*p* ≤ 0.05) and are represented on figures by annotated letters. Additionally, to compare the effect of tested NPs at the concentrations of IC_50_ and ½IC_50_, the independent Student’s *t*-test for the *p* < 0.05 was used. Furthermore, cluster analysis was applied to evaluate how closely associated NPs treatments are over the whole set of data. Principal component analysis (PCA) and the Pearson correlation coefficient (Pearson’s r; *p* ≤ 0.05) were calculated to determine the linear dependence of all variable values. All of the statistical studies were conducted using MS Office 2019 (Microsoft Inc., Redmond, WA, USA) and the STATISTICA 13.1 software package (TIBCO Software Inc., Palo Alto, CA, USA).

## 5. Conclusions

The results presented in this study confirmed the diverse influence of Ag-NPs, Cu-NPs, ZnO-NPs and TiO_2_-NPs on the expression level of selected genes and the activity of their secondary molecular counterparts in *E. coli*, *B. cereus* and *S. epidermidis* cells. The effect of NPs on the gene expression level depended on the type and concentration of NPs and the species of bacteria. Despite the considerable diversity of the results, it turned out that, in most cases, the regulation of the expression of selected genes was correlated with the activity of the encoded proteins, especially those with CAT and PER-type activities. Moreover, the obtained results confirmed the ability of bacterial cells to respond to stress caused by NPs, providing protection against oxidative stress. Undeniably, the conducted study is innovative, as it provides direct evidence in the explanation of the biological action of metal and metal oxide NPs at the molecular level. The presented results are valuable, as they confirm the ability of the tested bacterial strains to activate sophisticated and diverse strategies of defence against ROS in order to minimise oxidative damage.

## Figures and Tables

**Table 1:** Primer sequences for selected housekeeping and oxidative stress genes in *E. coli*, *B. cereus* and *S. epidermidis* used in the RT-qPCR reaction.

Gene/Product	Sequence of Primer		Product Length [bp]
	Forward (5′-3′)	Reverse (5′-3′)	
***E. coli***			
**Housekeeping genes**			
*gyr*A (DNA gyrase subunit A)	CTTCATCGAATAGACGCGG	TCTGCCGCACGTATTAAAG	108
*gyr*B (DNA gyrase subunit B)	GCAAAGAAGACCACTTCCAC	AAGATATTCGGGTGGATCGG	91
*rpo*E (RNA polymerase sigma-E factor)	TACAGCAATCCGATACAGCC	TCCCGATGTGGTACAAGAAG	100
**Oxidative stress genes**			
*kat*E (CAT HPII)	CATTCGGGAGTAGAGCAGTT	ATGATGAAGTGAGATCGGCA	89
*kat*G (bifunctional CAT-PER)	ACGTAAATCAGGCCCATCTC	TCTGGATGTTAACTGGGGTG	105
*ycd*B (heme-containing PER)	CGTAATCGGGAACATCATGC	TGAAAGAGCAGCAGACGATA	87
*sod*A (manganese SOD)	TTATCGCCTTTTTGCACCAG	GCTATCGAACGTGATTTCGG	110
*sod*B (cytosolic iron-containing SOD)	CTTCAGCGACTTTTCCAGTC	TCTGAAGGTGGCGTATTCAA	109
*sod*C (copper-zinc SOD)	AAGCTGCCAGTGAAAAAGTC	GTTTCAGTAATGGTGACGCT	88
***B. cereus***			
**Housekeeping genes**			
*gyr*A (DNA gyrase subunit A)	TACGTTGGGCGATGAAGACC	AATCGGTGTACGCTTTCCGT	103
*gyr*B (DNA gyrase subunit B)	GCGTGGTATTCCGGTTGGTA	TATAACCGCCACCGCCAAAT	104
*rpo*B (RNA polymerase subunit beta)	ACCAGAGGGACCAAACATCG	CTGGGTCAACACGACGGTAT	101
**Oxidative stress genes**			
*kat*A (main CAT)	CAACAACGTGATGGTGCGAT	GTTGAATCGCGGTAAGCTGG	110
*kat*E (CAT HPII)	GGCCCAACCTTAATGGAGGA	TAACCATGTACGCCAACCCC	110
*tpx* (thiol PER)	GCGCTGATTTACCATTCGCTC	GAATGAAAGGTCGCGGTGGT	92
*yoj*M (zinc SOD-like protein)	GAAGGGTGCAGAAAACGGTG	TCAAGTGTGATGTGTGGGGC	93
*sod*A1 (manganese SOD)	CCAGAAGCAATCCGTACAGC	CTCCGCCGTTTGGAGATAGG	91
*sod*A2 (manganese SOD)	CGAAATAACGGTGGTGGTCA	TGCAACGTCTCCATTAGGCT	90
***S. epidermidis***			
**Housekeeping genes**			
*gyr*B (DNA gyrase subunit B)	GACAATGGCCGTGGTATTCCT	CCGAATTTACCTCCAGCGTG	98
*rpo*B (RNA polymerase subunit beta)	GGGAGCAAACATGCAACGTC	TCTCTTGCGGCTACGTGTTC	90
*pyk (*pyruvate kinase)	ACTGCTGGTGTACCTACTGGA	CCTCTACCAACACCTTGACCT	98
**Oxidative stress genes**			
*bsa*A (glutathione peroxidase homolog)	CGCTGCTAAAGGTATGTAAACGA	TCCGGTTTCTTTTGAGGGGAG	108
*kat*A (CAT)	AGTCGTGATGGACAAATGCG	GTGGCTTCTTGTGTTCAGGC	109
*npr* (NADH peroxidase)	CCAGCTACCGAGTGGCTAAA	GCCACCCGCATAGACATCTT	105
*tpx* (thiol PER)	ACGCTTACTTGCACGTTCGG	CAGGGTAATTCGTACCTTCGCT	89
*sod*A (SOD Mn/Fe)	TCAGCAGTGAAGGGACAGATT	CCACCGCCATTATTAGAACAG	110

**Table 2:** The IC_50_ and ½IC_50_ values of tested NPs against bacterial strains [40].

Type of NPs (Size, nm)	*E. coli*		*B. cereus*		*S. epidermidis*	
	Toxicological Parameters [mg L^−1^]					
	IC_50_	½IC_50_	IC_50_	½IC_50_	IC_50_	½IC_50_
Ag-NPs (<100)	7.84	3.92	480.10	240.05	442.20	221.10
Cu-NPs (25)	180.80	90.40	52.15	26.075	112.00	56.00
TiO_2_-NPs (20)	43.40	21.70	50.30	25.15	703.40	351.70
ZnO-NPs (<50)	176.10	88.05	319.10	159.55	201.70	100.85

## Data Availability

Not applicable.

## Supplementary Materials

The following supporting information can be downloaded at: https://www.mdpi.com/article/10.3390/ijms23094966/s1.
