# Supplementary material for: Evaluation of the Effects of Ag, Cu, ZnO and TiO2 Nanoparticles on the Expression Level of Oxidative Stress-Related Genes and the Activity of Antioxidant Enzymes in Escherichia coli, Bacillus cereus and Staphylococcus epidermidis
Source: Int J Mol Sci. 2022 Apr 29;23(9):4966. doi: 10.3390/ijms23094966 (PMC9103769; doi:10.3390/ijms23094966)
Supplement: Supplementary file 1 [file ijms-23-04966-s001.zip › ijms-1680150-supplementary.pdf]

Table S1. Pearson's correlation matrix for enzyme activity and relative gene expression level in *E. coli* under NPs treatment.

	CAT	PER	SOD	*katG*	*katE*	*ycdB*	*sodA*	*sodB*	*sodC*
**CAT**	1.000	0.950*	0.858	0.657	0.454	0.674	-0.271	0.058	0.621
**PER**	0.950*	1.000	0.965*	0.820	0.618	0.832	0.012	0.200	0.782
**SOD**	0.858	0.965*	1.000	0.942*	0.796	0.948*	0.259	0.426	0.917*
katG	0.657	0.820	0.942*	1.000	0.945*	0.998*	0.540	0.678	0.997*
katE	0.454	0.618	0.796	0.945*	1.000	0.944*	0.676	0.881*	0.968*
ycdB	0.674	0.832	0.948	0.998*	0.944*	1.000	0.523	0.679	0.995*
sodA	-0.271	0.012	0.259	0.540	0.676	0.523	1.000	0.755	0.569
sodB	0.058	0.200	0.426	0.678	0.881*	0.679	0.755	1.000	0.735
sodC	0.621	0.782	0.917	0.997*	0.968*	0.995*	0.569	0.735	1.000

CAT – catalase activity; PER – peroxidase activity; SOD - superoxide dismutase activity; *katG*, *katE*, *ycdB*, *sodA*, *sodB*, *sodC* - relative gene expression level; *level of significance $p < 0.05$.

Table S2. Pearson's correlation matrix for enzyme activity and relative gene expression level in *B. cereus* under NPs treatment.

	CAT	PER	SOD	*katA*	*katE*	*tpx*	*yojM*	*sodA1*	*sodA2*
**CAT**	1.000	0.872	0.853	0.526	0.528	0.816	0.929*	0.852	-0.187
**PER**	0.872	1.000	0.888*	0.417	0.649	0.774	0.882*	0.744	-0.058
**SOD**	0.853	0.888*	1.000	0.147	0.267	0.493	0.737	0.638	0.214
katA	0.526	0.417	0.147	1.000	0.520	0.811	0.487	0.318	-0.234
katE	0.528	0.649	0.267	0.520	1.000	0.849	0.783	0.743	-0.710
tpx	0.816	0.774	0.493	0.811	0.849	1.000	0.892*	0.784	-0.493
yojM	0.929*	0.882*	0.737	0.487	0.783	0.892*	1.000	0.960*	-0.466
sodA1	0.852	0.744	0.638	0.318	0.743	0.784	0.960*	1.000	-0.612
sodA2	-0.187	-0.058	0.214	-0.234	-0.710	-0.493	-0.466	-0.612	1.000

CAT – catalase activity; PER – peroxidase activity; SOD - superoxide dismutase activity; *katA, katE, tpx, yojM, sodA1, sodA2* - relative gene expression level; *level of significance $p < 0.05$.

Table S3. Pearson's correlation matrix for enzyme activity and relative gene expression level in *S. epidermidis* under NPs treatment.

	CAT	PER	SOD	*bsaA*	*katA*	*npr*	*tpx*	*sodA*
**CAT**	1.000	0.918*	0.976*	-0.268	0.065	-0.164	-0.186	-0.230
**PER**	0.918*	1.000	0.879*	-0.403	-0.019	-0.326	-0.351	-0.366
**SOD**	0.976*	0.879*	1.000	-0.393	-0.109	-0.295	-0.340	-0.363
bsaA	-0.268	-0.403	-0.393	1.000	0.912*	0.993*	0.946*	0.998*
katA	0.065	-0.019	-0.109	0.912*	1.000	0.936*	0.908*	0.931*
npr	-0.164	-0.326	-0.295	0.993*	0.936*	1.000	0.962*	0.997*
tpx	-0.186	-0.351	-0.340	0.946*	0.908*	0.962*	1.000	0.959*
sodA	-0.230	-0.366	-0.363	0.998*	0.931*	0.997*	0.959*	1.000

CAT – catalase activity; PER – peroxidase activity; SOD - superoxide dismutase activity; *bsaA*, *katA*, *npr*, *tpx*, *sodA* - relative gene expression level; *level of significance $p < 0.05$.
